# Intercalated architecture of Mg_2_AlXY_5_ monolayer with built-in potential difference and high-power-conversion efficiencies

**DOI:** 10.1016/j.isci.2025.114225

**Published:** 2025-11-26

**Authors:** Lili Liu, Yuanpeng Yang, Huimin He, Cai Chen, Xuelin Zhang, Mohamed Sharaf, Xiaozhi Wu

**Affiliations:** 1 College of Teacher Education, Chongqing Three Gorges University, Chongqing 404100, People's Republic of China; 2 School of Civil Engineering, Chongqing Three Gorges University, Chongqing 404100, People's Republic of China; 3 Institute of Sustainable Industries and Liveable Cities, Victoria University, Melbourne, VIC, Australia; 4 Department of Physics Institute for Structure and Function, Chongqing University, Chongqing 401331, People's Republic of China

**Keywords:** Applied sciences, Energy storage

## Abstract

Screening novel two-dimensional (2D) layered materials that combine high stability with strong power-conversion efficiency has attracted considerable attention owing to their promise in 2D optoelectronic devices. However, centrosymmetric structures are often not conducive to the separation of photogenerated-carriers. Therefore, we propose a strategy to design a non-centrosymmetric multi-atomic layer monolayer, namely, Mg_2_AlXY_5_ (X = Ga, In; Y = S, Se, Te) using first-principles calculations. The results demonstrate that these Mg_2_AlXY_5_ monolayers possess excellent structural stability and built-in potential difference, which can effectively promote the separation of photogenerated carriers. Moreover, most of them exhibit desirable direct band gaps and high electron mobilities (up to ∼10^3^ cm^2^V^−1^s^−1^), indicating optical absorption spanning the near-infrared to visible region. Interestingly, spin-orbit coupling (SOC) drives an indirect-to-direct band-gap transition in Mg_2_AlGaTe_5_ and Mg_2_AlInTe_5_ monolayers. In addition, the Mg_2_AlGaSe_5_ monolayer is an effective donor material, and the corresponding Mg_2_AlGaSe_5_/InSe type II heterostructure achieve outstanding power-conversion efficiencies of 18.64%.

## Highlights

• New strategy to architect non-centrosymmetric Mg_2_AlXY_5_ monolayers
• Strong optical absorption from the near-infrared to the visible region
• High electron mobilities (up to 3.36 × 10^3^ cm^2^V^−1^s^−1^)
• A suitable donor material with the highest power-conversion efficiency of 18.64%

## Graphical abstract

**Figure:**
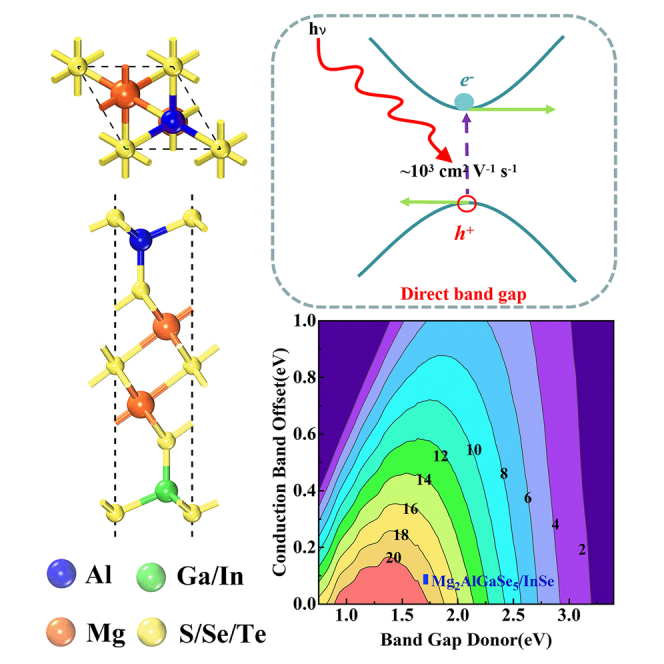

## Introduction

Two-dimensional (2D) layered materials have attracted widespread interest owing to their unique geometries, high carrier mobilities, and tunable band gaps.^1^^,^^2^^,^^3^^,^^4^^,^^5^^,^^6^^,^^7^ Meanwhile, the exciton binding energies of monolayer semiconductors are typically an order of magnitude greater than those of three-dimensional bulk materials, primarily due to weakened Coulomb screening arising from reduced dimensionality.^8^^,^^9^^,^^10^^,^^11^ Notably, recent advances in synthesis have yielded multicomponent layered materials with excellent stability.^12^^,^^13^^,^^14^^,^^15^^,^^16^^,^^17^^,^^18^ Therefore, the rational design of novel 2D monolayers with desirable band gaps and high carrier mobilities is highly promising for applications in energy storage, gas sensing, and optoelectronic devices.

Recently, an increasing number of two-dimensional (2D) ternary layered materials have been experimentally realized. For example, ultrathin (∼2.4 nm) layered PbSnS_2_ with highly anisotropic optoelectronic properties has been synthesized via chemical vapor deposition (CVD).^19^ Layered compounds with high stability and carrier mobility, such as SnPS_3_, CdPSe_3_, and Bi_2_O_2_X, exhibit excellent optical properties, making them promising candidates for optoelectronic applications.^20^^,^^21^ Moreover, several 2D quaternary layered materials (i.e., LiInP_2_S_6_ and CuInP_2_S_6_) have been synthesized,^22^^,^^23^ displaying low ionic conductivity and outstanding photocatalytic CO_2_ reduction, respectively.^24^^,^^25^ In addition, seven-atomic-layered (Mo/W)Si_2_N_4_ and MnBi_2_Te_4_ have been experimentally realized and have attracted extensive attention due to their compelling optoelectronic properties and phenomena, such as the quantum anomalous Hall effect.^26^^,^^27^^,^^28^^,^^29^^,^^30^ Nine-atomic-layer Mg_2_Al_2_Se_5_-like monolayers have been proposed, exhibiting excellent power-conversion efficiency.^31^ Notably, the nine-atomic-layer Mg_2_Al_2_Se_5_ monolayer comprises a bilayer 1T-phase MgSe_2_ combined with a single layer of Al_2_Se_2_. It is well known that rapid separation of photogenerated carriers is a key factor in enhancing photoelectric conversion efficiency; however, symmetric monolayer structures are generally unfavorable for effective carrier separation, leading to reduced conversion efficiency. Fortunately, the nine-atomic-layer Mg_2_Al_2_Se_5_-like monolayer can serve as an ideal model for constructing non-centrosymmetric monolayer structures.

Therefore, based on first-principles calculations, we propose a strategy to design a non-centrosymmetric monolayer family Mg_2_AlXY_5_ (X = Ga, In; Y = S, Se, Te). First, the lattice stability of these Mg_2_AlXY_5_ monolayers is established via phonon spectra and *ab initio* molecular dynamics (AIMDs) simulations at 300 K. We then systematically analyze their electronic structures, optical absorption spectra, and electron mobilities, which together indicate excellent light-absorption efficiency and high electron transport capability. In particular, these nonpolar Mg_2_AlXY_5_ monolayers exhibit a built-in potential difference that effectively promotes the separation of photogenerated carriers. Furthermore, the Mg_2_AlGaSe_5_ monolayer is identified as a suitable donor material, enabling the construction of type II heterojunctions for solar cell applications.

## Results and discussion

**Figure 1:**
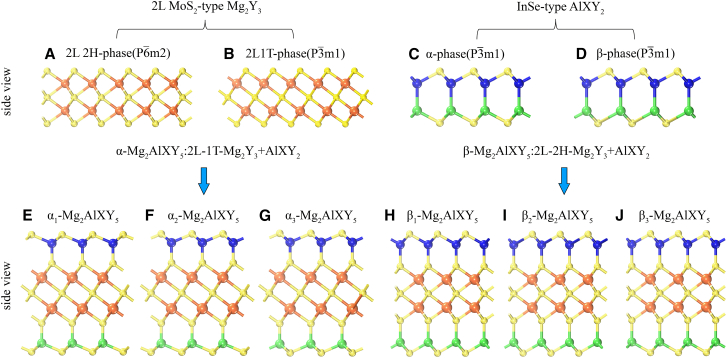
Schematic diagram of the intercalated process of performing the bilayer (A) Double-layer 2H-phase MoS_2_-type (Mg_2_Y_3_). (B) Double-layer 1T-phase MoS_2_-type (Mg_2_Y_3_). (C) Single-layer α-phase InSe-type (AlXY_2_). (D) Single-layer β-phase InSe-type (AlXY_2_). (E–G) Intercalated α-Mg_2_AlXY_5_ monolayers. (H–J) Intercalated β-Mg_2_AlXY_5_ monolayers.

**Figure 2:**
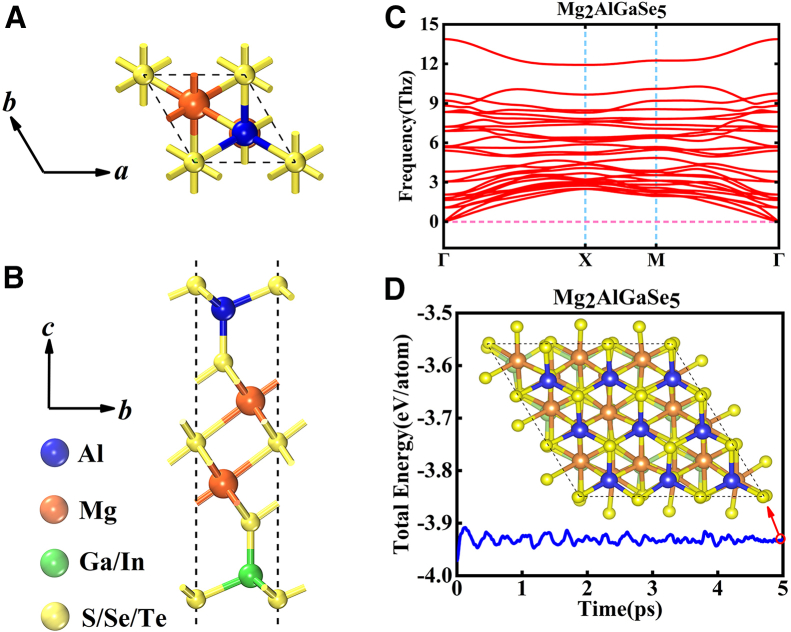
Schematic diagram of Mg_2_AlXY_5_ monolayer and the analysis of stability (A) The top view of Mg_2_AlXY_5_ monolayers. (B) The side view of Mg_2_AlXY_5_ monolayers. (C) The phonon spectra of Mg_2_AlGaSe_5_ monolayers. (D) The AIMD (300 K) of Mg_2_AlGaSe_5_ monolayers.

Since layered Mg_2_Al_2_Se_5_ has been synthesized experimentally, we predict a new family of nine-atomic-layer Mg_2_AlXY_5_ monolayers, constructed by stacking a bilayer 1T- or 2H-phase Mg_2_Y_3_ (Y = S, Se, Te) with an α- or β-phase AlXY_2_-like monolayer (Figure 1). We propose six possible polymorphs, denoted α_1_-Mg_2_AlXY_5_, α_2_-Mg_2_AlXY_5_, α_3_-Mg_2_AlXY_5_ and β_1_-Mg_2_AlXY_5_, β_2_-Mg_2_AlXY_5_, β_3_-Mg_2_AlXY_5_ (Figures 1E–1J). After full optimization, the α-phase Mg_2_AlXY_5_ monolayers are significantly lower in energy than their β-phase counterparts, with α_1_-Mg_2_AlXY_5_ being the most stable (Figure 1E). Taking Mg_2_AlGaS_5_ as an example, we compare the total energies of the different phases in Figure S1. Accordingly, we investigate the compositions Mg_2_AlGaS_5_, Mg_2_AlGaSe_5_, Mg_2_AlGaTe_5_, Mg_2_AlInS_5_, Mg_2_AlInSe_5_, and Mg_2_AlInTe_5_. The nine-atomic-layer Mg_2_AlXY_5_ monolayer comprises nine sublayers: two-atom-thick AlY (or XY) layers at the bottom and top surfaces and a central bilayer 1T- and 2H-phase Mg_2_Y_3_ block (Figures 2A and 2B). Notably, this architecture yields nonpolar Mg_2_AlXY_5_ monolayers that nevertheless exhibit an intrinsic built-in potential difference arising from the chemical asymmetry between Al and X (X = Ga, In).

We first report the lattice constants of the Mg_2_AlXY_5_ (X = Ga, In; Y = S, Se, Te) monolayers, as listed in Table 1, and these nine-atomic-layer Mg_2_AlXY_5_ systems adopt a hexagonal lattice. We then systematically assess their structural stability. On one hand, phonon spectra were computed using the finite-displacement method. Taking the Mg_2_AlGaSe_5_ monolayer as an example, no imaginary modes are observed throughout the Brillouin zone, indicating excellent dynamical stability (Figure 2C). On the other hand, AIMD provides an effective evaluation of thermodynamic stability. Accordingly, we performed AIMD at 300 K for the Mg_2_AlGaSe_5_ monolayer (Figure 2D). The total energy of the supercell exhibits only small fluctuations over time, and structural snapshots at 5,000 fs further confirm robust thermodynamic stability. Phonon spectra and AIMD results for the remaining nine-atomic layer Mg_2_AlXY_5_ monolayers are provided in Figures S2 and S3, respectively, demonstrating outstanding structural stability across this 2D family.

**Table 1:** The lattice constants (*a*=*b*) and band gaps (*E*_g_) of these Mg_2_AlXY_5_ monolayers with PBE (*E*_g1_) and HSE06 (*E*_g2_) levels, and the SOC effect have been considered in Mg_2_AlGaTe_5_ and Mg_2_AlInTe_5_ monolayers

Structure	Lattice *a*=*b* (Å)	*E*_g_ (eV)	
		*E*_g_^PBE^	*E*_g_^HSE06^
Mg_2_AlGaS_5_	3.69	1.56	2.73
Mg_2_AlGaSe_5_	3.89	0.83	1.84
Mg_2_AlGaTe_5_	4.23	0.39	1.16
SOC-Mg_2_AlGaTe_5_	4.23	0.18	0.75
Mg_2_AlInS_5_	3.76	1.69	2.80
Mg_2_AlInSe_5_	3.96	1.11	2.11
Mg_2_AlInTe_5_	4.29	0.75	1.54
SOC-Mg_2_AlInTe_5_	4.29	0.52	1.15

**Figure 3:**
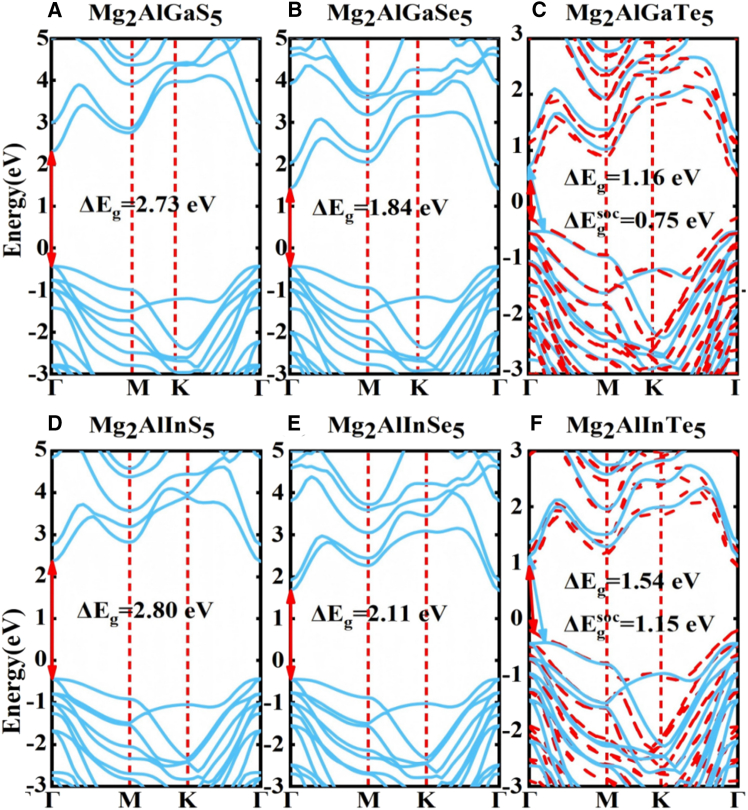
Band structures of Mg_2_AlXY_5_ monolayers by HSE06 level (A) The band structure of Mg_2_AlGaS_5_ monolayer. (B) The band structure of Mg_2_AlGaSe_5_ monolayer. (C) The band structure of Mg_2_AlGaTe_5_ monolayer with (red) and without (cyan) SOC, respectively. (D–F) (D) The band structure of Mg_2_AlInS_5_ monolayer. (E) The band structure of Mg_2_AlInSe_5_ monolayer. (F) The band structure of Mg_2_AlInTe_5_ monolayer.

**Figure 4:**
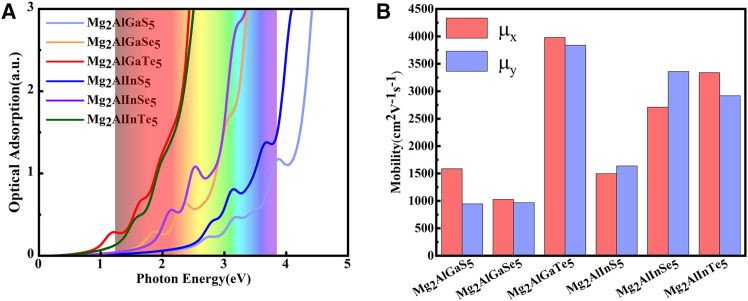
The absorption spectra and the electron mobilities of all Mg_2_AlXY_5_ monolayers (A) The optical absorption of Mg_2_AlXY_5_ monolayers with the HSE06 level. (“a.u.” stands for “%”). (B) The electron mobilities of Mg_2_AlXY_5_ monolayers.

Because the electronic structure is a key determinant of semiconductor performance, we systematically examined the band structures of the nine-atomic-layer Mg_2_AlXY_5_ monolayers. As expected, the semilocal Perdew-BurkeErnzerhof (PBE) functional underestimates the band gaps (Figure S4). We therefore employed the high-accuracy HSE06 hybrid functional to refine the electronic structures, and the corresponding results are shown in Figures 3A–3F. The valence-band maximum (VBM) and conduction-band minimum (CBM) of monolayer Mg_2_AlGaS_5_, Mg_2_AlGaSe_5_, Mg2AlInS5, and Mg_2_AlInSe_5_ are located at Γ, indicating direct gap semiconductors with the HSE06 band gaps of 2.73 eV, 1.84 eV, 2.80 eV, and 2.11 eV, respectively (see Table 1), which is substantially smaller than that of the Mg_2_Al_2_Se_5_-like monolayer and comparable to those of PbSnS_2_ and SnPS_3_ monolayers.^32^^,^^33^ By contrast, for Mg_2_AlXTe_5_ (X = Ga, In), the CBM remains at Γ while the VBM shifts off Γ to a point between Γ and M, yielding indirect-gap semiconductors with HSE06 gaps of 1.16 eV and 1.54 eV, respectively (see Table 1). Moreover, we assessed the influence of spin-orbit coupling (SOC) on the electronic structures at the PBE level (see Figure S4). Clearly, the SOC effect significantly alters the electronic structures of the Mg_2_AlGaTe_5_ and Mg_2_AlInTe_5_ monolayers, and their band gaps are reduced by more than 30% relative to the case without SOC due to the presence of the heavy element Te. We therefore examined SOC at the HSE06 level for these two systems. Notably, SOC drives an indirect-to-direct band-gap transition in both Mg_2_AlGaTe_5_ and Mg_2_AlInTe_5_, with the gaps reduced to 0.75 and 1.15 eV, respectively (Figures 3C and 3F). Moreover, we analyzed the orbital contributions from the orbital-resolved electronic band structure of the Mg_2_AlGaSe_5_ monolayer (see Figure S5). The CBM arises primarily from the *s* orbitals of Al and S atoms, whereas the VBM is dominated by the *p*_x_ and *p*_y_ orbitals of S. Furthermore, HSE06 level optical absorption spectra for the nine-atomic-layer Mg_2_AlXY_5_ monolayers (Figure 4A) show absorption peaks predominantly in the near-infrared to visible region, in agreement with the band-gap results.

**Figure 5:**
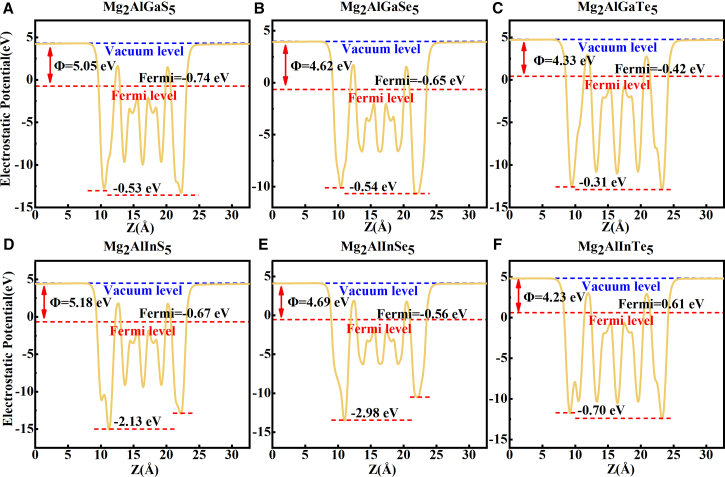
The effective electrostatic potential distribution along the Z direction of all Mg_2_AlXY_5_ monolayers (A) The effective electrostatic potential distribution of Mg_2_AlGaS_5_ monolayer. (B) The effective electrostatic potential distribution of Mg_2_AlGaSe_5_ monolayer. (C) The effective electrostatic potential distribution of Mg_2_AlGaTe_5_ monolayer. (D–F) (D) The effective electrostatic potential distribution of Mg_2_AlInS_5_ monolayer. (E) The effective electrostatic potential distribution of Mg_2_AlInSe_5_ monolayer. (F) The effective electrostatic potential distribution of Mg_2_AlInTe_5_ monolayer. The position of Fermi level, the work functions and the corresponding built-in potential difference caused by the difference between Al and X atoms are marked respectively.

Rapid separation of photogenerated carriers is a key determinant of photoelectric conversion efficiency. In general, non-centrosymmetric multicomponent monolayers possess intrinsic built-in electric fields that enhance light-absorption efficiency. Accordingly, we propose nine-atomic-layer Mg_2_AlXY_5_ monolayers that are overall nonpolar because the two surfaces are identically terminated. Nevertheless, the absence of inversion symmetry produces an internal potential difference across the layer due to the chemical asymmetry between Al and X (X = Ga, In), which effectively promotes electron-hole separation. The calculated potential differences for these Mg_2_AlXY_5_ monolayers span 0.31–2.98 eV (Figures 5A–5F). Consistent with the larger electronegativity contrast between Al and In relative to Al and Ga, the Mg_2_AlInY_5_ series exhibits substantially larger potential steps than the Mg_2_AlGaY_5_ counterparts (see Figure 5).

Carrier mobility plays a crucial role in assessing the optoelectronic properties of 2D semiconductors. Accordingly, we evaluated the carrier mobilities of the Mg_2_AlXY_5_ monolayers using the deformation-potential (DP) method,^34^^,^^35^ and computational details are provided in the supplemental information. The key parameters, including the DP constant *E*_l_, in-plane elastic modulus *C*_2D_, and effective mass *m∗*, are listed in Table 2 at 300 K, and the corresponding fits are shown in Figures S6 and S7. The hole effective masses are substantially larger than the electron effective masses across this family, consistent with the curvature of the band edges (CBM and VBM). We therefore report electron mobilities only. The calculated values (Table 2; Figure 4B) show that most Mg_2_AlXY_5_ monolayers exhibit electron mobilities exceeding 10^3^ cm^2^V^−1^s^−1^. The largest electron mobility is up to 3.36 × 10^3^ cm^2^V^−1^s^−1^ for Mg_2_AlInSe_5_, which is comparable to MoSi_2_N_4_-like monolayers and approaching those of Mg_2_Al_2_Se_5_-like monolayers. For Mg_2_AlGaTe_5_ and Mg_2_AlInTe_5_, inclusion of SOC increases *m∗* and consequently reduces the electron mobility (see Table 2). Also, the structural complexity gives rise to numerous high-frequency optical phonon branches and these optical modes introduce additional carrier-scattering channels, which can markedly influence and limit carrier mobility.^36^^,^^37^^,^^38^ Taken together with their favorable electronic properties, such as direct band gaps, intrinsic built-in potential differences, and high electron mobilities, these Mg_2_AlXY_5_ monolayers emerge as promising candidates for high-performance optoelectronic devices.

**Table 2:** The parameters, including effective masses (*m∗*), DP (*E*_L_), elastic modulus (*C*_2D_), and calculated electron mobilities (*μ*), of these Mg_2_AlXY_5_ monolayers at 300 K (*x* = Zigzag, *y* = Armchair)

Carrier type	Structure	Effective masses		Deformation potential (eV)		2D elastic modulus (J·m^−2^)		Mobility (10^3^ cm^2^V^−1^s^−1^)	
		*m*_*x*_^∗^/*m*_e_	*m*_*y*_^∗^/*m*_e_	|*E*_l*x*_|	|*E*_l*y*_|	*C*_2Dx_	*C*_2Dy_	*μ*_*x*_	*μ*_*y*_
Electron	Mg_2_AlGaS_5_	0.24	0.20	7.28	9.44	201.49	166.76	1.59	0.95
	Mg_2_AlGaSe_5_	0.15	0.16	12.63	12.65	180.08	178.00	1.03	0.97
	Mg_2_AlGaTe_5_	0.12	0.13	7.25	7.09	144.01	145.06	3.98	3.84
	SOC-Mg_2_AlGaTe_5_	0.18	0.18	6.98	6.94	143.16	143.17	1.93	1.96
	Mg_2_AlInS_5_	0.27	0.28	6.22	5.83	203.06	203.23	1.50	1.64
	Mg_2_AlInSe_5_	0.17	0.17	6.78	6.22	177.10	182.69	2.71	3.36
	Mg_2_AlInTe_5_	0.13	0.15	7.09	7.08	137.41	137.19	3.34	2.92
	SOC-Mg_2_AlInTe_5_	0.17	0.18	6.97	6.97	138.41	138.51	2.04	1.93

The SOC effects are considered in Mg_2_AlGaTe_5_ and Mg_2_AlInTe_5_ monolayers.

Previous studies indicate that semiconductors with an appropriate direct band gap (∼1.50 eV) are suited as donor materials for solar cells.^33^ Accordingly, the Mg_2_AlGaSe_5_ monolayer, with a direct band gap of 1.84 eV, is a promising donor candidate. For practical implementation, the acceptor material should have both its CBM and VBM lower than those of the donor, and forming a type II heterostructure that facilitates efficient separation of photogenerated carriers. At the HSE06 level, and referenced to the vacuum level, the CBM and VBM of Mg_2_AlGaSe_5_ and InSe are −3.91 and −6.74 eV, and −3.86 and −5.70 eV, respectively, yielding desirable type II heterojunction.

**Figure 6:**
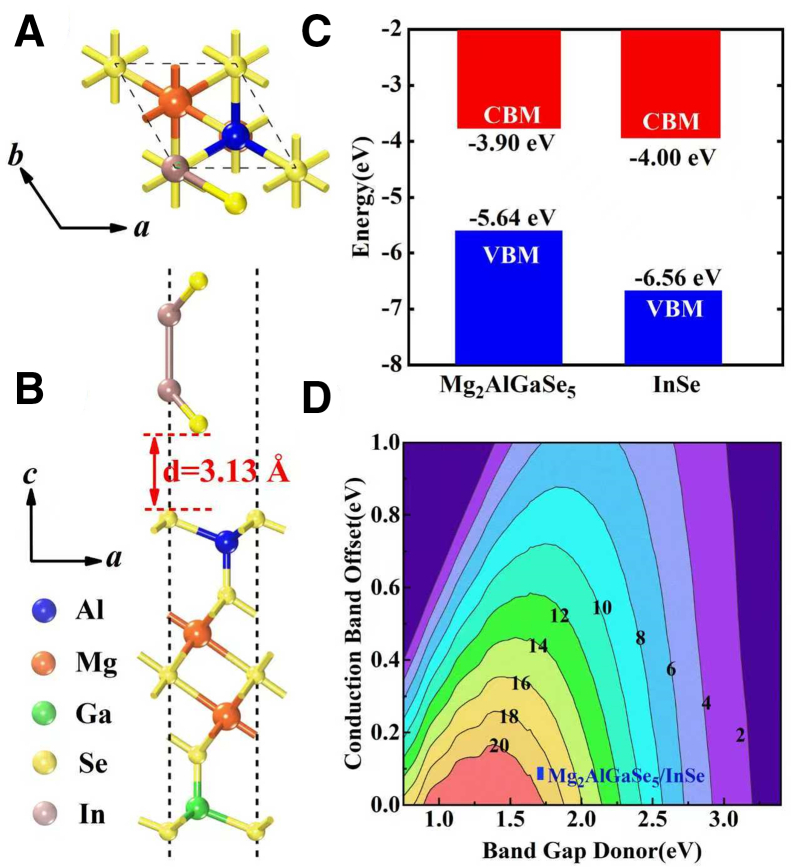
The structure of Mg_2_AlGaSe_5_/InSe heterostructure, the donor and acceptor band edge with corrected vacuum level, and the calculated PCE (A) The top view of Mg_2_AlGaSe_5_/InSe heterostructures. (B) The side view of Mg_2_AlGaSe_5_/InSe heterostructures. The Mg_2_AlGaSe_5_ and InSe are monolayers. (C) Schematic illustration of the corrected vacuum level donor and acceptor band edges of Mg_2_AlGaSe_5_ and InSe monolayers with HSE06 level. (D) The calculated PCE of Mg_2_AlGaSe_5_/InSe heterostructure.

Therefore, we have constructed six potential Mg_2_AlGaSe_5_/InSe type II heterostructures (see Figure S8) and screened one with the lowest energy for power conversion efficiency (PCE) calculation, as shown in Figures 6A and 6B. The corresponding lattice mismatches (for a single unit-cell match) is 3.7%. We calculated the electronic structure of the Mg_2_AlGaSe_5_/InSe vdW heterostructure at the HSE06 level and determined the CBM offset between the two constituents via vacuum-level alignment. The results show that the band gaps of Mg_2_AlGaSe_5_ and InSe^39^ decrease slightly, while the CBM offset increases (see Figure 6C), which we attribute to the small lattice strain and the weakened interlayer vdW coupling. Consequently, the predicted PCE is modestly adjusted to 18.64%, as shown in Figure 6D, which is comparable to those reported for Ca_2_Ga_2_Se_5_/ZrSe_2_, MoSi_2_P_4_/SIn_2_Te, and perovskite solar cells.^40^^,^^41^^,^^42^^,^^43^ Taken together, these results identify Mg_2_AlGaSe_5_/InSe type II heterojunctions as a promising candidate for solar cell applications.

In conclusion, based on the density functional theory, we propose a strategy to architect a non-centrosymmetric nine-atomic-layer monolayer Mg_2_AlXY_5_ (X = Ga, In; Y = S, Se, and Te). Phonon spectra and AIMD simulations demonstrate outstanding structural stability. Moreover, Mg_2_AlXY_5_ monolayers exhibit desirable direct band gaps, whereas Mg_2_AlXTe_5_ (X = Ga, In) displays indirect gaps. Notably, inclusion of SOC induces an indirect-to-direct band-gap transition in Mg_2_AlGaTe_5_ and Mg_2_AlInTe_5_. These monolayers show strong optical absorption from the near-infrared to the visible region and high electron mobilities (up to 3.36 × 10^3^ cm^2^V^−1^s^−1^). Importantly, an intrinsic built-in potential difference, arising from the chemical contrast between Al and X, can effectively promote the separation of photogenerated carriers. Furthermore, we identify the Mg_2_AlGaSe_5_ monolayer as a suitable donor material, and the corresponding Mg_2_AlGaSe_5_/InSe type II heterostructure achieve power-conversion efficiency of 18.64%. Overall, our findings provide a feasible route to architect non-centrosymmetric multicomponent monolayers and supply Mg_2_AlXY_5_ candidates for high-performance solar cells.^44^

### Limitations of the study

In this study, we calculated the electronic band structures and the optical absorption spectra of monolayer Mg_2_AlXY_5_ (X = Ga, In; Y = S, Se, and Te) by the HSE06 level. However, there is still a slight difference between the results calculated by the theoretical model and experimental value, although the corrected results are more accurate compared to PBE calculations. In order to obtain results close to the experimental value, the higher precision GW-(Bethe-Salpeter equation)-BSE calculations should be performed.

## Resource availability

### Lead contact

Further information and requests for resources and reagents should be directed to and will be fulfilled by the lead contact, Xiaozhi Wu (xiaozhiwu@cqu.edu.cn).

### Materials availability

This structure could be loaded in supplemental information.

### Data and code availability

• The simulation data that support the findings of this study are available from the lead contact upon reasonable request.
• This paper does not report original code.
• Any additional information required to reanalyze the data reported in this paper is available from the lead contact upon request.

## Acknowledgments

This work was supported by the Science and Technology Research Program of 10.13039/501100007957Chongqing Municipal Education Commission (grant no. KJZDK202301207), the 10.13039/501100001809National Natural Science Foundation of China (no.12174040), the Chongqing Natural Science Foundation (grant no. cstc2020jcyj-msxmX0118), and Chongqing Students innovation and entrepreneurship training program (no. 202510643007).

## Author contributions

L.L., writing – original draft, methodology, investigation, and conceptualization. Y.Y., investigation and data curation. H.H., investigation and data curation. C.C., writing – review & editing, project administration, investigation, and conceptualization. X.Z., investigation, methodology, and conceptualization. M.S., investigation and data curation. X.W., writing – original draft and formal analysis.

## Declaration of interests

The authors declare no competing interests.

## STAR★Methods

### Key resources table

**Table:** 

REAGENT or RESOURCE	SOURCE	IDENTIFIER
**Software and algorithms**		

VASP	VASP Software GmbH	https://www.vasp.at/
Material Studio software package	Dassault Systèmes BIOVIA	https://www.3ds.com/products/biovia/materials-studio
VESTA software package	JP-Minerals	https://jp-minerals.org/vesta/en/
Python 3.12	Python Software Foundation	https://www.python.org

### Method details

The Vienna Ab initio Simulation Package (VASP) was employed in this work.^45^^,^^46^^,^^47^ The generalized gradient approximation with the Perdew-BurkeErnzerhof (PBE) functional^48^^,^^49^ was used to describe the electron exchange-correlation effects, and the HSE06 hybrid functional^50^ was applied to refine the electronic structures. A plane-wave cutoff energy of 600 eV and a vacuum spacing of 20 Å were adopted. Structural optimizations used a Γ-centered 14×14×1 k-point mesh. The electronic energy was converged to 10^-6^ eV, and the maximum residual force on each atom was reduced below 0.01 eV Å^-1^. Ab initio molecular dynamics (AIMD) simulations at 300 K were conducted to assess the stability of the predicted 2D monolayers.

Moreover, the optical absorption coefficient is obtained by solving the following formulas:

$$\alpha \left(\omega \right)=\sqrt{2}\omega {\left(\sqrt{\varepsilon_{1}{\left(\omega \right)}^{2}+\varepsilon_{2}{\left(\omega \right)}^{2}}-\varepsilon_{1}\left(\omega \right)\right)}^{1/2}$$

Where the *α(ω)* represent the absorption coefficient, and the *ε*_1_*(ω)*, *ε*_2_*(ω)* represent the real parts and imaginary parts of the dielectric constants. The alignment of vacuum band energies is only carried out during the calculations of power-conversion-efficiency (PCE).
