# Supplementary material for: Intercalated architecture of Mg2AlXY5 monolayer with built-in potential difference and high-power-conversion efficiencies
Source: iScience. 2025 Nov 26;28(12):114225. doi: 10.1016/j.isci.2025.114225 (PMC12723374; doi:10.1016/j.isci.2025.114225)
Supplement: Document S1. Figures S1–S8 [file mmc1.pdf]

iScience, Volume *28*

Supplemental information

Intercalated architecture of Mg_2AlXY_5 monolayer with built-in potential difference and high-power-conversion efficiencies

Lili Liu, Yuanpeng Yang, Huimin He, Cai Chen, Xuelin Zhang, Mohamed Sharaf, and Xiaozhi Wu

Supplemental Information

Contents:

1. Supplemental Figures
2. Carrier Mobility Calculation
3. Power conversion efficiency calculation
4. POSCAR-(α-phase Mg_2AlGaS_5)
5. References

1. Supplemental Figures

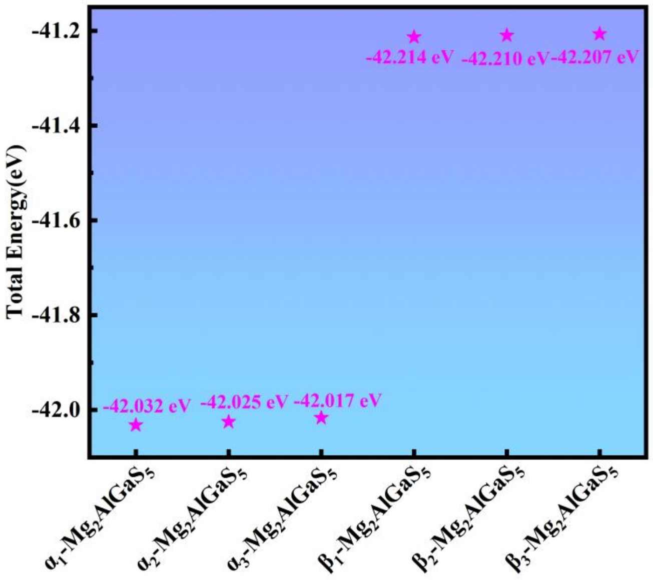

Figure S1. The total energies of different Mg_2AlGaS_5 monolayers.

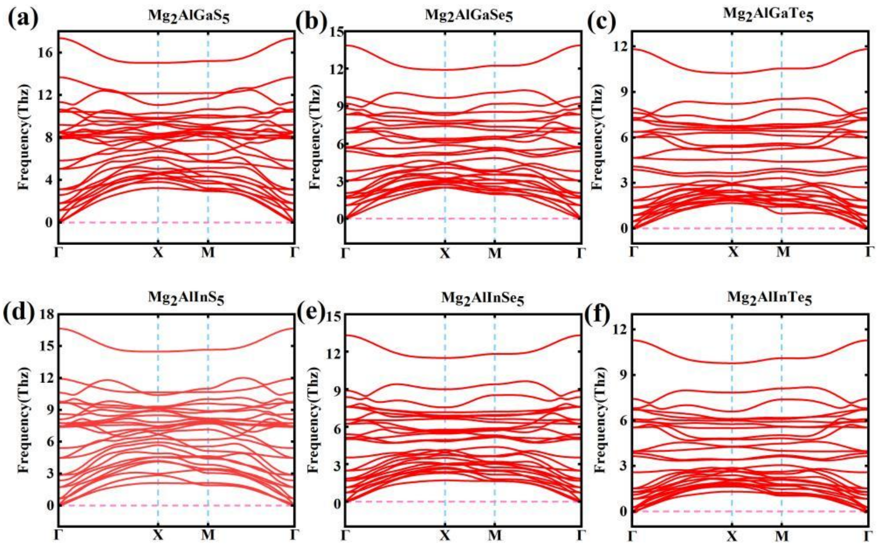

Figure S2. Phonon spectra of (a) Mg_2AlGaS_5, (b) $Mg_2AlGaSe_5$, (c) $Mg_2AlGaTe_5$, (d) Mg_2AlInS_5, (e) $Mg_2AlInSe_5$, and (f) $Mg_2AlInTe_5$ monolayers.

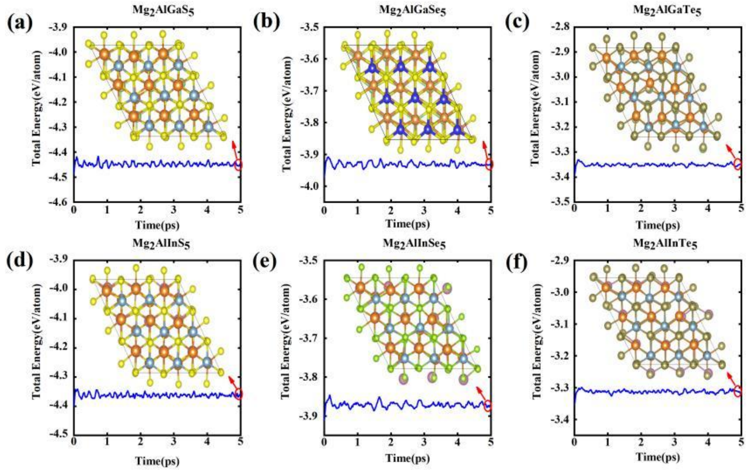

Figure S3. The total energies of (a) Mg_2AlGaS_5, (b) $Mg_2AlGaSe_5$, (c) $Mg_2AlGaTe_5$, (d) Mg_2AlInS_5, (e) $Mg_2AlInSe_5$, and (f) $Mg_2AlInTe_5$ monolayers as a function of time at 300 K, and the corresponding insets are the snapshots of the p(3 × 3) supercell of these six 2D monolayers at 5 ps.

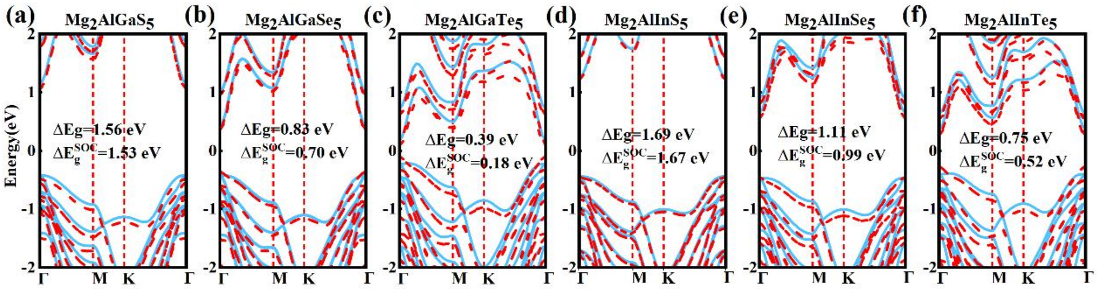

Figure S4. Band structures of Mg_2AlGaS_5, $Mg_2AlGaSe_5$, $Mg_2AlGaTe_5$, Mg_2AlInS_5, $Mg_2AlInSe_5$, and $Mg_2AlInTe_5$ monolayers with (red)/ without (cyan) SOC by PBE level, respectively.

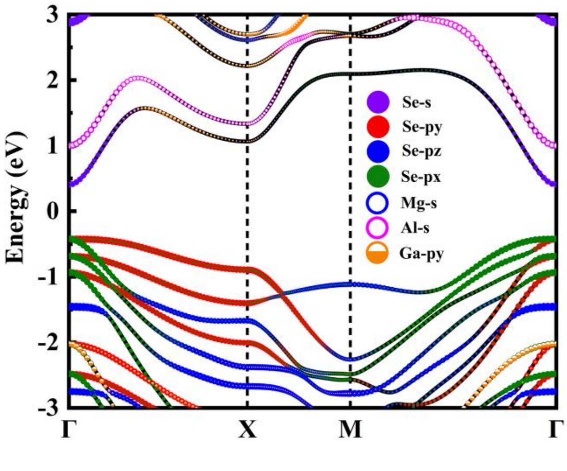

Figure S5. The orbital-resolved electronic band structure of $Mg_2AlGaSe_5$ monolayer.

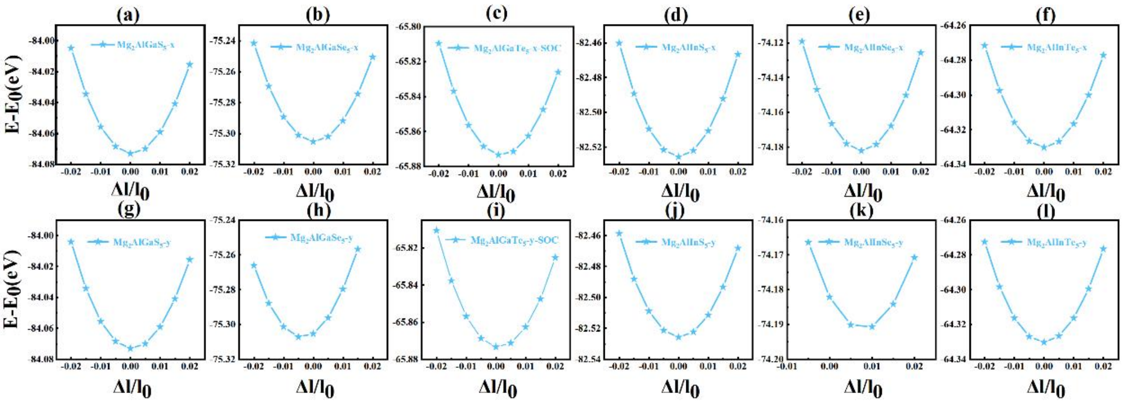

Figure S6. The energy difference between the total energy of monolayer Mg_2AlXY_5 (X = Ga, In; Y = S, Se, and Te) under uniaxial strain and that without strain as a function of the strain along *x* (zigzag) and *y* (armchair) directions, respectively. The in-plane stiffness C_{2D} can be obtained after fitting the parabola.

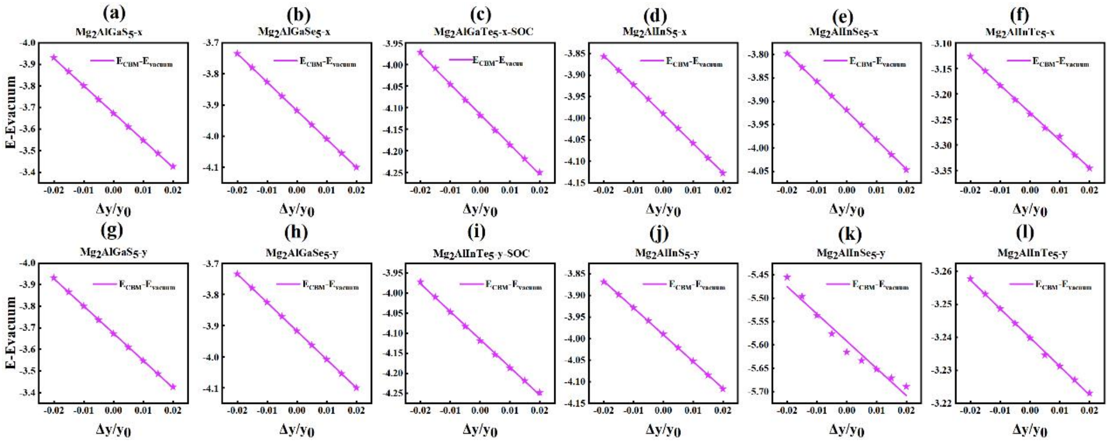

Figure S7. The energy difference between band energies of the CBMs of monolayer Mg_2AlXY_5 (X = Ga, In; Y = S, Se, and Te) and the vacuum energy as a function of the lattice dilation along *x* (zigzag) and *y* (armchair) directions, respectively. The slopes of the purple lines correspond to the DP (Deformation Potential) constant along with different directions for electrons.

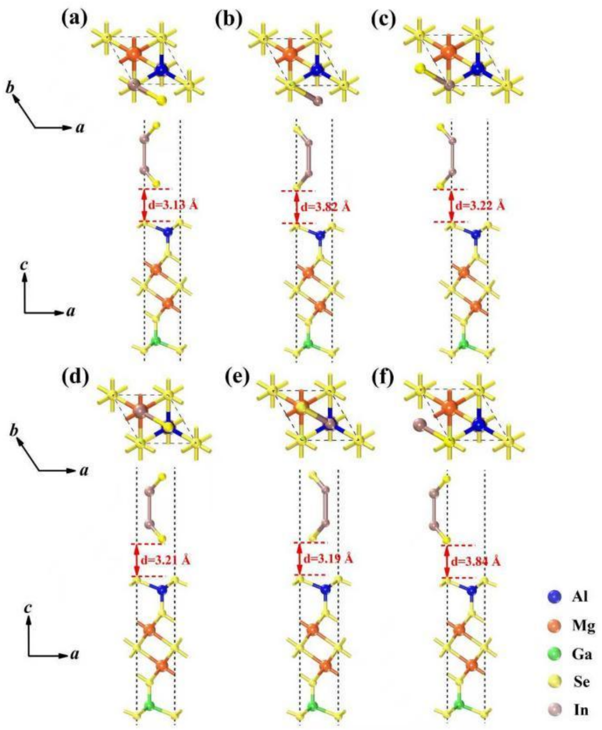

Figure S8. The top and side views of $Mg_2AlGaSe_5$/InSe heterostructures with six different stacking modes, where $Mg_2AlGaSe_5$ and InSe are monolayers.

2. Carrier Mobility calculation

To investigate the material electron and hole transport properties, we use the phonon-limited scattering model of the deformation potential theory proposed by Bardeen and Shockley to predict the carrier mobility of monolayer Mg_2AlXY_5 (X = Ga, In; Y = S, Se, and Te). For the 2D semiconductor system, we used the following equation to calculate the intrinsic carrier mobility of the monolayers[1,2]

$$\mu_{2D} = \frac{e\hbar^3 C_{2D}}{K_B T m^* m_d (E_l)^2},$$

where T, K_B, C_{2D}, E_l, m^* are the temperature, Boltzmann constant, elastic modulus of a uniformly deformed crystal, the variational constant along the transport direction, and the effective masses of electrons and holes, respectively. m^* depends on the energy change of the wave vector k along different transport directions, defined as $m^* = \hbar^2/(\partial^2 E/\partial^2 k)$. m_d is the average effective mass, which is determined $m_d = \sqrt{m_x m_y}$. The valence band maximum (VBM) of electrons alongthe transport direction or the conduction band minimum (CBM) of the deformation situationconstant E_l, determined by $E_x = \Delta E_{lx}/(\Delta_{lx}/_{lx0})$ and $E_y = \Delta E_{ly}/(\Delta_{ly}/_{ly0})$. The calculation of thedeformation potential C_{2D} and the distortion potential Elin the paper is based on the equilibriumlattice structure, respectively, along with the uniaxial axis of the in-plane cell or by applyingdifferent values of stretching and compression in the interval from -2.0% to 2.0%

(taking a series of discrete values at 0.5% intervals) to achieve the calculation of the cell under different strains.

3. Power conversion efficiency calculation

The power conversion efficiency (PCE) η can be evaluated at the limit of 100% external quantum efficiency (EQE) using the formula given by

$$\eta = \frac{J_{sc} V_{oc} \beta_{FF}}{P_{solar}} = \frac{0.65(E_g^d - \Delta E_c - 0.3)\int_{E_g}^{\infty} \frac{P(\hbar\varpi)}{\hbar\varpi} d(\hbar\varpi)}{\int_0^{\infty} P(\hbar\varpi) d(\hbar\varpi)},$$

where J_{sc} denotes the short circuit current in the limit of 100% EQE that can be calculated by the integral in the numerator, in which $P(\hbar\omega)$ is the AM1.5 solar energy flux (expressed in W m^2 eV^{-1}) at the photon energy $\hbar\omega$; V_{oc} is the maximum open circuit voltage estimated by ($E_g^d - \Delta E_c - 0.3$), in which E_g^d represents the band gap of the donor materials (monolayer $Mg_2AlGaSe_5$). Consequently, the PCEs of the $Mg_2AlGaSe_5$/CdS and $Mg_2AlGaSe_5$/InSe type-II heterostructures were obtained by calculating the horizontal coordinates (band gaps for the donor, E_g^d) and vertical coordinates (the difference in CBM between the donor and acceptor materials, ΔE_c). β_{FF} is the band-fill factor, which is generally set as 0.65; P_{solar} is the AM1.5 solar flux that can be evaluated by the integral in the denominator[3-6].

4. POSCAR-(α-phase Mg_2AlGaS_5)

```
MgAlGaS
1.00000000000000
3.6948277069311843    0.0000000000000000   0.0000000000000000
-1.8474249315321620   3.1998097357560615   0.0000000000000000
0.0000000000000000    0.0000000000000000   32.0680015564000016
S   Mg   Ga   Al
5    2     1    1
Cartesian
0.000002681    0.000007178    16.389905075
-0.000004937   2.133213992    13.549434237
-1.847422519   3.199806589    22.378061610
1.847411869    1.066605852    19.249618102
0.000011383    0.000005048    10.341004397
-0.000004387   2.133210178    17.902955928
1.847409722    1.066613004    14.873465550
0.000013232    2.133205982    11.361819876
1.847416274    1.066599749    21.409417033
```

5. References

1. Qiao, J., Kong, X., Hu, Z.-X., Yang, F., and Ji, W. (2014). High-mobility transport anisotropy and linear dichroism in few-layer black phosphorus. Nat. Commun. *5*, 4475. 10.1038/ncomms5475.

2. Bardeen, J., and Shockley, W. (1950). Deformation Potentials and Mobilities in Non-Polar Crystals. Phys. Rev. *80*, 72-80. 10.1103/PhysRev.80.72.
3. Adilbekova, B., Lin, Y., Yengel, E., Faber, H., Harrison, G., Firdaus, Y., El-Labban, A., Anjum, D.H., Tung, V., and Anthopoulos, T.D. (2020). Liquid phase exfoliation of MoS_2 and WS_2 in aqueous ammonia and their application in highly efficient organic solar cells. J. Mater. Chem. C *8*, 5259-5264. 10.1039/d0tc00659a.
4. Kim, H.D., Yanagawa, N., Shimazaki, A., Endo, M., Wakamiya, A., Ohkita, H., Benten, H., and Ito, S. (2017). Origin of Open-Circuit Voltage Loss in Polymer Solar Cells and Perovskite Solar Cells. ACS Appl. Mater. Interfaces *9*, 19988-19997. 10.1021/acsami.7b03694.
5. Ehrler, B., Alarcón-Lladó, E., Tabernig, S.W., Veeken, T., Garnett, E.C., and Polman, A. (2020). Photovoltaics Reaching for the Shockley–Queisser Limit. ACS Energy Lett. *5*, 3029-3033. 10.1021/acsenergylett.0c01790.
6. Herterich, J., Baretzky, C., Unmüssig, M., Maheu, C., Glissmann, N., Gutekunst, J., Loukeris, G., Mayer, T., Kohlstädt, M., Hofmann, J.P., and Würfel, U. (2022). Toward Understanding the Short-Circuit Current Loss in Perovskite Solar Cells with 2D Passivation Layers. Sol. RRL *6*. 10.1002/solr.202200195.
